# Paradigms and Public Policies on Drought in Northeast Brazil: A Historical Perspective

**DOI:** 10.1007/s00267-015-0444-x

**Published:** 2015-01-21

**Authors:** José Nilson B. Campos

**Affiliations:** Departamento de Engenharia Hidráulica e Ambiental, Universidade Federal do Ceará, Campus do Pici, Bloco 713, Fortaleza, Ceará 60451.970 Brazil

**Keywords:** Public policy, Drought, Semiarid, Water management, Hydrologic infrastructure

## Abstract

This paper describes the evolution of drought-related public policies in Northeast Brazil (NEB). Using a historical approach, we show that the evolution of public policy has not been characterized by abrupt shifts, but has instead been shaped through debates between renowned intellectuals. The resulting public policies formed a hydrological infrastructure that delivers clean water needed for robust economic activity. However, outcomes of the 2012–2013 drought show that populations that depend on rain fed agriculture are as vulnerable to drought as they were at the start of the 20th century. Although government, social, and emergency programs have aided drought victims, drought analysts agree that rain fed agriculture has remained vulnerable since drought policies were first formulated. Drought policies formulate integrated water resources management (IWRM) strategies that are geared toward supplying safe drinking water, and debates surrounding the IWRM paradigm have been affected by outcomes of major international events such as the World Water Forum.

## Introduction

Drought is an insidious phenomenon that characterizes climates in virtually all regions of the world (Wilhite and Buchanan-Smith 2005). Historically, droughts have been associated with famine and mortality in Brazil, China, and India and in roughly seven to eight African countries (Kebbede and Jacob 1988; Davis 2002; Endfield and Tejedo 2006). In other countries such as Australia, the United States, and England, droughts have been associated with economic losses in agriculture (Heathcote 1991, 1991; All 2006; Chiew et al. 2011; Ummenhofer et al. 2009). In both cases, public policy development generally requires significant public investment, thus spurring debates in the media and in academic communities.

Scientists now play a more active role in policy development, and especially in cases of complex issues such as drought management (O’Meagher et al. 1998). These researchers stress the importance of scientific input in political decision making to improve drought management systems, and key studies have examined the evolution of drought policies in Australia and South Africa, which address highly variable arid climatic conditions.

Reactive government approaches to drought relief are common worldwide and are often less financially efficient. Immediately following the 1985 famine in Ethiopia, the government invested heavily in an ambitious program, though a large portion of funds were ultimately wasted or allocated inefficiently (Hoben 1995).

Nevertheless, some drought relief programs have been deemed successful by scientists. After Bostwana gained independence, the federal government initiated an agricultural relief and recovery program from 1992 to 1996 that managed livestock resources and emergency human drinking water supplies. This drought relief program has been deemed relatively successful (Belbase and Morgan 1994).

While the drought of 2000 in Ceará State affected the Brazilian economy only marginally, it affected a large number of people. In 2000, while agriculture accounted for only 5.58 % of the state’s GDP, it employed 40 % of the state’s labor force, largely through small, rain fed subsistence farming (Chimeli et al. 2008).

Through a case study of Kenya’s Drylands, Eriksen and Lind (2009) conveyed drought adaptation as a political process. The authors found that in times of drought, relationships are forged between individuals, politicians, institutions, and government administrations that aim to retain or strengthen political power and secure material means of survival. The authors also note that national economic and political processes significantly affect local adaptive capacities of regional resource allocation, policy development, and elected official competition.

“Big” questions are those that have “large-scale outcomes that are regarded as substantively and normatively important by both specialists and nonspecialists” (Mahoney and Rueschemeyer 2003, p. 7). Northeast Brazil (NEB) droughts were considered a “big” question during the 1877–1879 drought in Brazil. This drought occurred during a period marked by climatic disasters that caused millions of deaths in India, China, and Brazil (Davis 2002).

Historical analyses are needed to examine such “big” questions. In this paper, public policies are analyzed based on their treatment of six issues: (1) the treatment of climatic adversity; (2) the collection of drought-related information; (3) the development of hydrological infrastructure; (4) the generation of ecological perspectives; (5) the improvement of economic profiles; and (6) the support of sustainable development (SD) and integrated water resources management (IWRM).

This paper focuses on issues of drought. More specifically, it examines the evolution of public policies and paradigms over more than two centuries in the NEB. Studies focusing on drought typically examine relative short and acute drought events. This study is novel in its historical examination of the evolution of public policies on drought and of debates held among scientists and politicians in the media and in major scientific events. This analysis of trends over two centuries offers a summary of ideas and paradigms on drought issues in NEB. The study thus provides a historical profile of drought events that have occurred in the NEB, where droughts are considered a so-called “big” question among the regional population.

## Methods and Study Area

### Climate and Environment

NEB is divided into six well-defined eco-regions: the semiarid back lands (*sertões*); the coastal humid forest zone (*zona da mata*); a savannah ecosystem in the western area of the region (*agreste*), which is a transitional zone between *zona da mata* and *sertões*; the pre-Amazonian transition zone; and several microclimates within the *sertões.* The semiarid eco-region accounts for up to 60 % of the region and includes an area north of the state of Minas Gerais (Magalhães and Glantz 1992, p. 17).

The semiarid eco-region is the driest region of Brazil. Annual precipitation levels range from 400 to 800 mm, and evaporation levels reach over 2,000 or 3,000 mm in some areas. Frequent and intense droughts characterize this region. Most of the local soils cover a crystalline substrate, which when combined with the local rainfall regime generates intermittent rivers in the northern area of the region.

The rainfall regime occurs over a few months, with the majority of precipitation falling from February to May. Farmers plant at the start of the humid season (February to March) and harvest at the end of the season (May to June).

**Fig. 1:**
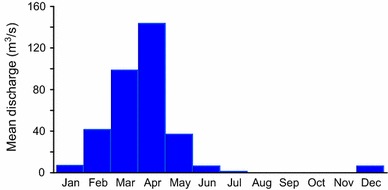
Monthly mean discharge from the Jaguaribe River in Iguatu, Ceará State

The regional river regime is unique. The majority of riverine discharge is generated over 3 months (Fig. 1). During longer drought events, these rivers may remain dry for up to 18 months. To address this problem, reservoir construction policies were implemented. These structures use water collected during humid periods to alleviate water shortages during drought periods. These reservoirs serve as a safe drinking water source for economic activities.

**Fig. 2:**
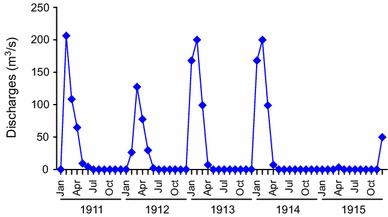
Daily discharge from the Jaguaribe River from January of 1912 to December of 1915

Figure 2 presents daily discharge levels for the Jaguaribe River in Iguatu from January of 1912 to December of 1915. This river remained dry or nearly dry for 18 months from June of 1914 to November of 1915. Given limited underground water reserves under natural conditions, it is impossible to support large cities in addition to water-dependent economic activities. In response to this vulnerability, reservoirs have been built that can store water during high discharge periods for use during droughts.

**Fig. 3:**
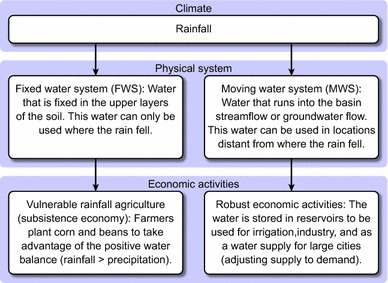
Environmental and social systems of water use in Northeast Brazil

A representation of ways in which local populations have adapted to local climate conditions is shown in Fig. 3. Rainfall is used as the primary water source for household, agricultural, and economical uses.

When rainfall reaches the soil, a portion remains fixed in the upper soil layer while the rest runs off the surface or enters the underground flow. Earth systems that receive this water can be defined as follows: (1) fixed water systems (FWS) installed within water-retaining soil layers, wherein water is only used where rainfall occurs; and (2) mobile water systems (MWS) that utilize rivers, reservoirs, aquifers, and lakes. MWSs are used to provide safe drinking water for economic activities (Campos and Studart 2008).

FWS systems are vulnerable to droughts that affect rain fed agriculture. Such droughts result from insufficient or irregular rainfall distributions that cannot maintain sufficient soil moisture during periods longer than vegetative crop cycles. Such droughts have the greatest social impact on rural populations of semiarid regions.

### Research Methodology

Case studies are useful in cases where existing theoretical or statistical methods are weak (George and Bennet 2005). George and Bennet (2005) highlight four advantages of case studies for theory development: conceptual validity; new hypothesis generation; the ability to examine the hypothesized role of causal mechanisms in the context of individual cases; and the capacity to examine causal complexities.

This study examines the evolution of policies in three areas: public policies, ideas of key intellectuals, and paradigms. The policy study presents historical facts corresponding to the period from the 1877–1879 drought to the 2012–2013 drought. Historical public policy research refers as much as possible to original sources and relies to a great extent on works on Mossoró of the Rio Grande do Norte by Vinght-et-Un Rosado, who compiled several historical documents on drought in a collection of 16 books. This collection is available at the Federal University of Ceará library.

## The Evolution of Public Policies and Paradigms

Climatic patterns shape the environment, and societies adapt to the environment. In turn, public policies directed toward the development of buildings and hydraulic infrastructure change physical systems, requiring societies to again adapt to modified conditions. Changes in these interdependent dimensions delineate the evolution of water management models used by societies, and this has been the case in NEB. The advancement of public policies and economic development of semiarid regions reflect societal attempts to adapt to changing natural and modified environments. This section presents paradigms, public policies, and ideas discussed by historical leaders since the start of colonization.

### Early Climatic Adversity: Before Public Policy Formation

The first recorded narrative on NEB drought is that of Jesuit Priest Fernão Cardim, who arrived in Bahia, Brazil in 1583. Cardim traveled along the Brazilian coast from Pernambuco to Rio de Janeiro. Throughout his journey, Cardim wrote an epistolary narrative on NEB drought. He describes a drought that occurred in 1583 as follows:

In 1583, there was such intense drought and sterility in the Province (a rare event because the area generally receives continuous rain) that water mills stopped grinding for a long time. There was a great famine, mainly in the *sertões* of Pernambuco, and four or five thousand Indians uprooted from *sertões* due to starvation moved to the coast seeking assistance from Caucasian populations.^1^

The Pernambuco coast is located in the *Zona da Mata*, which is characterized by continuous precipitation. Data show that in 1583, there was low precipitation along the Pernambuco coast and a drought strongly affected the *sertões.* From this testimony, one can infer that the semiarid region is naturally vulnerable to drought, and even in the presence of low population densities, as was the case at that time.

As settlements formed in the *sertões*, local droughts took on a new quality. Official documents indicate considerable losses in farming production during droughts. An official document published at the start of the 18th century shows how courts and colonizers viewed droughts in the region. Alves (2003) transcribed a letter sent by local officers to the Portuguese king:

The Camera Official that represents the King stated that from 1723 to the present (1729), the region has suffered from considerable sterility due to drought. He requested that the King assist him in sending additional slaves as many slaves had died from starvation, and he explained that mills were in ruins not only due to dry soils, but also due to insufficient manpower.

Two conclusions can be deduced from this excerpt: (1) droughts heavily affected the rudimentary economy forming in the region, and (2) a culture of landlords appealing to government to solve drought-related economic problems formed during the colonial period.

### On the Origins of Knowledge and Ignorance on Drought

This sub-section addresses how Brazilian intellectuals and politicians viewed issues and resources pertaining to the distant northern region. The first institutional initiative to gather information on NEB was led by the Historical and Geographical Brazilian Institute (IHGB). At a session chaired by Emperor D. Pedro II on May 30th, 1856, all members signed a proposition to create a commission of Brazilian engineers and naturalists dedicated to exploring lesser-known Brazilian provinces (Braga 1961).

Two members of the imperial commission were involved in debates concerning NEB droughts: Guilherme de Capanema and Raja Gabaglia.

#### Gabaglia held peculiar views on this issue:

Providence was bountiful, but man was wasteful. Notorious improvidence has characterized forest conservation; forests, now almost virtually destroyed, have become scrublands or caatingas, which are shrubs. The land now supports little vegetation as a result of neglect and destruction. Overall, the following conditions are evident to the lesser observer: climatic conditions harmful to human life and agricultural production, poor road transportation, forest abandonment and destruction, and streams with barely moistened streambeds (Gabaglia 1985, p. 11).

Capanema (1985) held a similar perspective, arguing that droughts resulted from the improvidence of those who did not know how to effectively manage soils in drought conditions. In fact, for these key intellectuals, droughts were not an obstacle to regional development. Rather, slow economic growth in the region was attributed to agricultural workers themselves. Nevertheless, others viewed droughts as a huge obstacle to overcome.

Engineer Viriato de Medeiros participated intensely in debates on drought. Responding to reservoir, channel, and reforestation proposals and unfounded theories, he declared:

1) Droughts cannot be mitigated by humans, as they are governed by predetermined atmospheric laws. 2) No artesian well, channel, forestation or reservoir solutions would minimize or prevent droughts. 3) The best means of minimizing extraordinary droughts, which are periodic, involves developing an understanding of periods in which they occur based on meteorological observation (Medeiros 1985).

Thus, for Medeiros, droughts were inevitable, and proposed solutions were largely ineffective. He proposed instead building several meteorological stations to observe and record all phenomena, and mainly those related to dominant winds. According to him, it was possible to forecast droughts 2 or 3 months before they began and to move populations and livestock to more secure regions. Medeiros also proposed the formation of government emergency programs.

Rohan disagreed with Medeiros’s ideas regarding the inefficiency of reservoirs. He listed three potential benefits of constructing reservoirs in NEB:

1) Water would be available throughout Ceará Province and even during drought periods, unlike at present; in turn, populations and cattle would not suffer anymore. 2) Reservoirs would serve as place for the propagation of several species of fish, which would become an important resource for local populations. Currently, when rivers dry up, fish populations, confined within small pools of water, die in the mud […]. 3) Reservoirs would attract waterfowl and riparian species and thus reinvigorate wildlife, which would serve as a valuable resource for the local population (Rohan 1985).

Nevertheless, Rohan held overly optimistic views of the benefits of reservoirs and forests. He expected that large-scale reservoir construction together with reforesting would transform NEB into a humid region.

The Polytechnic Institute of Rio de Janeiro also engaged in these debates and held numerous seminars on drought in NEB. From these seminars, the imperial government formed a commission dedicated to identifying practical methods of supplying water in Ceará.

This government action was motivated in response to public clamor resulting from the Great Drought, which resulted in hundreds of thousands of casualties. American journalist Smith (1879) estimated the number of deaths resulting from this event while conducting research in Brazil. According to him:

Roughly 500,000 residents died in Ceará from 1877 to 1878, accounting for more than half of the population. Among these casualties, 50,000 died of hunger and disease during the first year, and 50,000 died during in January and February of 1878. In March and April of 1878, the period of the great exodus, at least 150,000 people died, mainly of hunger. Fever and beriberi killed 100,000 people and chickenpox killed 80,000 or more. The remaining deaths resulted from various illnesses, primarily linked to hunger, weakness and malnutrition.

These statistics are likely greatly inflated by Smith’s shocked response to the disaster. Nonetheless, the tragic 1877 drought event serves as a landmark illustration of how drought issues became framed as a national problem over the views of Raja Gabaglia and Capanema. The central focus of debates thus changed to the following: how are the effects of drought best mitigated? Hence, the issue of drought in NEB became elevated as a “big question.”

### Hydraulic Solutions to Drought: The First Paradigm

Certainly, for a region where all rivers remain dry for more than 6 months each year, politicians first planned to accumulate water in reservoirs to guarantee year-round water access. Reservoir construction was thus the first approach used to solve NEB drought issues. Public policies were characterized by this approach from the start of drought public policy formulation in 1877 until the early 1950s.

Several countries have addressed water shortage issues through strategies that involve increasing water supplies. Such strategies have been referred to as hydraulic missions (Allan 2003), “high modern style” policymaking (Swatuk 2008), and “water resources engineering” (Magalhães, and Glantz 1992).

The Brazilian government began to construct reservoirs to serve as safe sources of water in 1879. The Ministry of Agricultural Affairs formed a commission to evaluate the feasibility of reservoir construction in Ceará. Engineer Jules Jean Revy arrived in Ceará in 1880 to manage the construction of a dam at the Cedro site in Quixadá. Though project planning concluded in 1882, construction did not start until 1884. Construction work was halted and reactivated several times thereafter until the dam’s completion in 1906.

Hydrological infrastructure policies became more effective in 1909 with the creation of the *Inspetoria de Obras Contra as Secas* (IOCS). The first general director of the IOCS, engineer Arrojado Lisboa, developed institutional action guidelines that remained in place until 1950. According to Lisboa:

Drought, in a strictly lexical sense, refers to a lack of moisture. From the rain comes water needed to support life on Earth. Drought problems, from this perspective, are thus simply viewed as water problems, or as issues of water supply. However, drought occurring in territory inhabited by humans has a more complex meaning. In fact, the physical phenomenon of rainfall scarcity affects man via modifications that affect economic conditions of the region, which, in turn, are reflected in the social order (Lisboa 1984).

Lisboa viewed drought as a multifaceted problem, and not as a purely engineering problem. For him, drought-inducing climatic conditions and rainfall scarcity trigger economic and social problems. Lisboa did not believe that infrastructure construction could solve these problems alone. He placed special emphasis on educational approaches:

We have arrived at the most pressing problem: the education problem. Only education will improve human health and teach individuals irrigation practices, to tailor industrial production to environmental characteristics, to use hay and silage, to not abandon cattle, to improve breeds, to build small reservoirs, and to understand, at last, the importance of efforts conducted for their welfare.

Lisboa also knew that technical and scientific knowledge was essential to form effective public policies. During his term, the IOCS contracted American scientists to study the region. The resultant report illustrated complex features of the drought-affected region from geographic, geologic, climatic, botanic, and economic perspectives. This indicates that, in Lisboa’s view, knowledge of the region was scarce and that there were few Brazilian experts that studied the region. The commissions produced several key reports that were used to establish drought management policies.

In the first half of the 20th century, infrastructure in NEB improved significantly. Several reservoirs, roads, and railroads were constructed, and many small cities were formed close to newly built reservoirs. Nevertheless, droughts in the *sertões* continued to challenge the population, which were sustained by rudimentary agriculture based on scarce rainfall.

### The Ecological Perspective: An Emergent Paradigm

Even after more than a half century of reservoir construction, droughts still significantly affected rural families. In response, scientists introduced new ways of addressing NEB droughts.

An ecological approach to the issue was first proposed by agronomy engineer Guimarães Duque, who had dedicated his professional life to research on the Brazilian semiarid region. One of his areas of focus examined *sertanejo* population livelihoods in adverse climatic conditions. For Duque:

Human survival is only possible when the biome occupies an advanced evolutionary state and is maintained there without degradation and when agriculture is managed without destroying the ecological balance or regular stability with fluctuations and repercussions of forms of life in the environment (Duque 1953).

Duque was also concerned about the limits of natural resource exploration:

Interdependence between soil, vegetation and climate has placed limits on agricultural work. These natural limits restrict uses of natural resources. Disrespecting this unwritten Natural Code will have repercussions, sooner or later, subtle or serious, depending on the intensity of transgressions performed.

According to a modern-day interpretation, Duque’s words reflect his reservations of development and agricultural exploration models when applied to semiarid regions. In essence, he was advocating for SD:

When pressures on the Drought Polygon reach a certain limit, a fraction of the population emigrates. Growing populations cause this human displacement, rupturing the biotic equilibrium between habitat availability and essential life needs either periodically or through a drought, quickly diminishing environmental sustainability. Human societies in dry regions live in fragile biotic harmony with the soil, flora and fauna despite cultural misunderstanding of symbiotic ecological interdependence between living things. Though the Northeast region has not yet reached its demographic economic capacity, it is marching toward saturation (Duque 1953).

In this excerpt, Duque notes the limits that regional drought-prone conditions impose on growth. It is interesting to observe that the rural population of Ceará had reached 2,015,846 by 1950, growing to a maximum of 2,105, 843 in 2000 and then decreasing to 2,105,824 in 2010^2^. Therefore, Ceará’s rural population in 2010 was basically the same as it was at the time of Duque’s writings.

Present in Duque’s works are concerns about education, and specifically of knowledge on climate and nature. Duque and Lisboa share similar views on development issues in Brazilian semiarid regions. Nevertheless, despite Duque’s role as a scientific leader in NEB, this paradigm was not effectively realized through public policy. The state was thus still “one step ahead” in the realm of reservoir policymaking.

### The Economic Development Paradigm

This paradigm is synonymous to the paradigm of high modernity “where the state and private sector activities, assisted by developments in science and technology, gave shape to the hydraulic mission” (Swatuk 2008). For Allan (2003), this forms the *second paradigm* of industrial modernity. Rather, the “ideas of the Enlightenments, engineering capacity science and the investment initiatives of the state and the private sector characterized industrial modernity” (Allan 2003, p. 13).

This paradigm persisted in NEB from the end of 1950’s to the start of 1980’s (Campos 2014). Its main purpose was to create development poles that attract rural families vulnerable to drought. As in the paradigm industrial modernity, this paradigm involves using reservoir water to support economic stability in NEB. This approach was championed by economist Celso Furtado.

Celso Furtado had experienced periods of drought throughout his childhood. He completed a Bachelor’s of Law and postgraduate studies in Economics at Sorbonne University in Paris.

Furtado, like Lisboa and Duque, recognized problems associated with societal adaptation to semiarid environments, or, as described by Duque, societal misunderstandings of the environment. However, Furtado was more critical of this maladaptation. For him, economic systems in semiarid regions served as “one of the most egregious cases of divorce between man and the environment, between life systems and regional ecological characteristics” (Furtado 1959).

Hydrological and road infrastructure systems were more comprehensive during Furtado’s time than during Lisboa’s time. Several new cities and economic activities had been established. Large cities such as Fortaleza were served by mid-sized reservoirs such as the Acarape reservoir, which was built in 1924. Thus, a portion of the semiarid region maintained economic activities that were supported by a robust, reservoir-based water system.

However, rain fed agriculture vulnerability remained largely unchanged since the start of the century. When droughts occurred, peasants could not always subsist from local economic activities or savings. The 1958 drought displaced hundreds of thousands of people. As at the start of the century, *sertanejos* built roads in work groups using primitive techniques. However, the extent of government assistance had improved. Vaccinations were administered, and water trucks were used to draw water from reservoirs for public consumption. In turn, deaths from starvation, thirst and disease, such as typhoid fever, plummeted.

During the 1958 drought, political misuse of drought and corruption in the application of public resources became well known in the public domain. Drought-related water policies were heavily criticized.

Hence, drought issues needed to be addressed with a new strategy. A new paradigm was thus spearheaded with the formation of the Economic Development Policy for the Northeast, formulated in 1958 under the management of Celso Furtado. In turn, the Brazilian government created the *Superintendência de Desenvolvimento do Nordeste* (SUDENE) in 1959 to plan development in the Northeast region. The SUDENE first planned industrialization in the region and re-organized public policies.

Furtado, like Arrojado Lisboa, turned to foreign consultants to improve scientific and technological expertise in the region. SUDENE hired technical experts from Israel, France, and Germany to study water resources and soils and to construct irrigation mechanisms that use reservoir water.

Large irrigation districts were formed to convert *sertanejos* from rainfall-based agricultural systems to systems of irrigated agriculture. The objective was thus to move communities from dependence on vulnerable FWSs to robust MWSs.

### Sustainable Development (SD) and Integrated Water Resource Management (IWRM) Paradigms

In the 1980s, scientific debates redefined water issues as “multi-dimensional, multi-sectorial, and multi-regional.” From these debates “emerged a new paradigm of Integrated Water Resources Management as the internationally preferred option for both developed and developing countries” (Agyenim and Gupta 2012, p. 47). The concept of SD also emerged during this decade.

SD and IWRM paradigms influenced NEB drought policies as a result of the state of changing international paradigms. This sub-section describes IWRM and SD paradigms in NEB drought public policies.

#### IWRM *Paradigms* in Semiarid Policies

For Allan (2003), this was a reflexive phase characterized by three major paradigms. Allan’s *third paradigm* concerns the changing of water allocation and management priorities. Allan’s fourth paradigm was inspired by economists and intended to draw the attention of water users in the North to the economic value of water and to its importance as a scarce economic resource. The main principles of this IWRM project involved *water valuation, water pricing, water markets, and privatization.* The notion that water allocation and management are political processes supports Allan’s *fifth paradigm.*

The Ceará State Water Master Plan (PERH) applied IWRM economic principles of water valuation and pricing. Public participation and the prioritization of water supplies needed for human and animal consumption were considered in Ceará’s Water Law. However, despite being discussed in several debates on IBRD loans, the Ceará State Water Management Model did not considered other water markets or water sector privatization.^3^ Changes in water allocation and management priorities (Allan’s third paradigm) were incorporated into the PERH in addition to the notion that water allocation and management are political processes.

The PERH had two foci: (1) developing a robust water management system for reservoir water; and (2) modeling an institutional system based on IWRM. The PERH conducted several studies to identify critical drought-affected areas. These reports concluded that while drought-related issues were not forgotten, they were no longer considered a major public policy concern.

Studies on the development of efficient reservoir systems were conducted as part of IWRM initiatives (Campos 2010). Global circulation models and regional atmospheric models were combined with hydrologic models to improve reservoir system efficiency (Alves et al. 2012).

#### SD in Semiarid Regions

International approaches to SD were effectively incorporated into public policies on drought in semiarid regions through the ARIDAS project of 1994 (Ministério do Planejamento e Orçamento 1995). ARIDAS was a collaborative effort between federal and state governments and non-governmental organizations that was geared toward building a SD model for Brazilian semiarid regions. ARIDAS was also the first structured study that evaluated effects of climate change on the environment and on societies of Brazilian semiarid regions.

ARIDAS examined two types of drought: edaphic and hydrological. Edaphic drought is related to soil dryness, causing crop losses in rain fed agricultural systems. Hydrological drought is associated with low reservoir water supplies available for drinking water use.

ARIDAS proposed various practices to reduce edaphic drought vulnerability, including the use of specific seed species, the use of climate forecasts for the determination of planting periods, the maximization of soil moisture-retention capacities, the use of fewer crops with less water-demanding vegetative cycles, and the adoption of rain-harvesting techniques. Nevertheless, these measures were largely insufficient in enabling farmers to generate surplus crops for use in inevitable drought periods (Campos 1997).

In essence, ARIDAS recognized the vulnerability of rain fed agriculture and challenges in developing technologies that cater this form of agriculture to semiarid conditions.

#### *Brazilian Social* Programs for Poverty and Rural *Family Health*

In 1997, Mexico initiated the Progresa program, a system that distributes cash to female leaders of poor households in exchange for regular child attendance at school, thereby improving diet, vaccination access, and health clinic attendance (Grinspson 2005).

Progresa’s success led to the development of the *Bolsa Família* program in Brazil (Campello and Neri 2013), which was created in 2003 along with the unification of various other social benefits. This program strives to benefit all Brazilians living in poverty and assists a large portion of the drought-affected population.

To specifically assist low-income rural populations plagued by drought, the Brazilian government created the *Bolsa Estiagem* program. Rural families already included in *Bolsa Familia* program are eligible for both benefits. Another social program catered to the drought region is the *Garantia Safra* initiative, which offers insurance to farmers that is paid during drought years (i.e., crops losses of over 50 %).

The Brazilian Army additionally performs the *operação carro*-*pipa* service, which involves collecting water from reliable sources for water tank truck distribution to rural families.

## Discussion

From 2012 to 2013, a severe drought (the worst in the last 30 years according to the meteorological institution of Ceará State (FUNCEME)) affected NEB. This event left large populations vulnerable to drought. However, in several ways, societal impacts differed from those historically characteristic of the region. The (2012–2013) drought caused fewer social disturbances than previous droughts, such as supermarket looting incidents or protests at municipal government facilities. This stability stemmed from government social programs.

This discussion reflects on the evolution of public policies and on their impacts on vulnerable rural NEB populations. The discussion covers four topics: drought relief programs and their impacts on vulnerable rural populations; program impacts on livestock; the evolution of debates among intellectuals and central paradigms; and impacts of water supply shortages on large cities and economic activities. In examining the evolution of public policies, features of the great drought of 1877–1879, 1958 drought, and 2012–2013 drought are compared and discussed.

### Evolution of Drought Relief Programs and Impacts on Populations

An estimated 500,000 people died in Ceará State as a result of the 1877–1879 drought, representing roughly half of the state population. Public work organization and coordination was very precarious. By December of 1878, 15,000 workers were employed in public works, representing a small proportion of the refugee population. The majority of refugees were housed in overcrowded camps, barely protected by huts or palm leaf shelters. Smith (1879, p. 419) compared photos of these refugees with photos of Andersonville prisoners published during the American Civil War. He also observed the misuse of public resources, even though only limited funds were involved. According to him, several relief commissioners responsible for administering limited funds allocated by the central government were “incompetent men and some were palpably dishonest” (Smith 1879, p. 413).

By 1958, NEB’s population reached 22.4 million inhabitants, with 14.7 million occupying the rural zone. Between 12 and 13 million people were affected by the 1958 drought (Robck 1963). Among this group, 550,000 were sent to work on public projects, primarily in road and small earth dam construction using very simple techniques (Pinheiro 1960). Although a large number of people were affected, comparisons between 1877–1879 and 1958 droughts show that public policies in practice for half a century had substantially reduced drought impacts.

Nevertheless, a negative outcome of the 1958 drought was the misuse of public resources by politicians for electoral purposes. The term *indústria das secas* (drought industry) developed prior to the drought was often used in the media. The term refers to the appropriation of collective resources intended for the well being of poor, semiarid rural families who depend on rain fed agriculture.

Qualitatively, from 2012–2013, drought impacts on rain fed agriculture were virtually the same as those in the past. Farmers experienced agricultural production losses and were unable to support themselves independently. From 2012–2013, 1,328,962 drought-affected people received benefits from the *bolsa estiagem* program (Ministério da Integração Nacional 2013), and 660,000 farmers received *garantia safra* benefits. Because recipients of *garantia safra* benefits did not receive *bolsa estiagem* resources, approximately two million people were direct beneficiaries of the drought relief program. Historically, these individuals would have been relegated to public works.

Compared to impacts of the 1958 drought, current public policies have delivered significant social benefits, mitigating drought effects considerably. Individuals are no longer employed in precarious public works projects, which are easily destroyed by first rains. Similarly, small cities are no longer flooded with rural populations in search of food and employment. This finding agrees with Barnet’s (2006) proposition that societal adaptation not only involves environmental and/or developmental measures, but also issues of peace and justice.

Such trends occur within a broader context of global climate variability. In spite of responses to climate variability, losses due to climatic hazards such as droughts continue to limit agricultural productivity, aggravating poverty levels (Eakin 2000).

### Evolution of Drought Impacts on Livestock

Historically, droughts have limited cattle production in *sertões.* The worst record of cattle production loss in Brazil occurred during the 1777 drought, when an estimated 7/8 of all cattle in the region died (Alves 2003). In response to such conditions, households sell off herds before livestock die or feed them various cactus species. For wealthier ranchers, it is not uncommon “to transport their livestock to other states or areas less affected by the drought” (Finan and Nelson 2001). It should be noted that in 1877, Viriato de Medeiros (see Sect. 3.2) proposed the use of drought forecasting to organize the transport of livestock to places unaffected by drought.

The death of cattle represented a turning point in the 2012–2013 drought. The media published images of dead cattle along roads. The majority of small reservoirs used for livestock had dried. Farmers attempted to prevent economic losses by moving cattle to areas where reservoirs were still full. Hence, the strategies used to minimize livestock losses were the same as those used a century before; nevertheless, hydrologic infrastructure that had been built created more options for cattle transportation.

Transporting cattle is risky under uncertain conditions. Seasonal forecasting is used as a modern approach under such conditions. Scientists use climate prediction to improve small farmer subsistence worldwide (Hansen 2002; Phillips et al. 2002; Grothmann and Patt 2005).

### Evolution of Debates on Drought Relief Programs

Prior to the 1877 drought, debates on the drought-affected population focused on the use of public resources for the provision of money and food. Two opposing points of view prevailed. Capanema (1985) believed that NEB farmers had caused their own misfortune and that the government must not spend money on drought policies. Viriato Medeiro argued that droughts were natural disasters and that the government should thus organize public works to help drought victims. Medeiros’s view prevailed during the 1877–1879 drought disaster, and thus public works policies were introduced. This framework remained for approximately one century.

Smith (1879, p. 418) proposed that money spent by the central government be used to provide honest work for the population in areas such as road construction and harbor improvement to prevent idleness. Nevertheless, the provincial government provided alms and daily rations. Ultimately, refugees asserted their right to this charity, and some lived in indolent inaction as a result.

However, Smith (1879) reported that in Baturité village, administrators provided food in exchange for labor. Only sick or old farmers were fed gratuitously. As in *Bolsa Familia*, children were required to attend school and learn to read and write before receiving food.

In becoming a “big question” issue, drought relief programs even entered Brazilian music and folklore. Immediately following the 1951–1952 drought, Luiz Gonzaga (1912–1989), the most famous singer of NEB regional music, performed a song written by Zé Dantas (1921–1962) named Voices of Droughts that describes a drought victim’s experience, stating that giving alms to a man who can work will either make one feel ashamed or transform one into a mendicant.

Debates concerning SD paradigms were settled in the context of international debates on poverty. Poor NEB farmers are included in the *Bolsa Familia* program based on conditional cash transfers. Current debates, according to Soares (2009), examine whether conditional cash transfers effectively reduce poverty levels.

### Water Suppl**ies** for Economically Robust Activities

There are two conceptual water supply systems within a region that includes intermittent rivers: a robust system with dependable water from large reservoirs; and a vulnerable system that relies on small reservoirs. Only a long sequence of droughts can affect a system of large reservoirs. Small reservoirs systems can typically sustain water from the wet season to the dry season; however, they cannot support 1 or 2 years of drought.

When the 1877 drought began, no large reservoirs were present in the region. All water supplied during the dry season was obtained from wells. While a small quantity of water could have been drawn from small reservoirs, these reservoirs were exhausted during droughts. Even wells in intermittent rivers with alluvium were exhausted during prolonged droughts. At this time, the economy was entirely based on agricultural activities with no industrial development in the region. Hence, the water crisis only affected rural populations.

In 1877, reservoir construction was dedicated to mitigating drought. The first large reservoir was completed in 1906, and policies to build reliable water supply infrastructure became effective only after the IOCS’s creation. By 1958, while several reservoirs served large cities in the region, farmers employed in public works projects did not have sufficient water access. Water supplies were transported by car tank from mid-sized to large reservoirs.

By 2012–2013, the number of large and medium reservoirs had increased significantly since 1958. The Metropolitan Region of Fortaleza, the Ceará State Capital, now has approximately 3.6 million inhabitants, representing 42 % of the state population. The area is served by a system of large reservoirs that include the Castanhão reservoir, the largest reservoir in Ceará. The region never experiences hydrologic drought, as the reservoir system can supply water for residents and state industries.

Nevertheless, populations that rely on water from small reservoirs remain vulnerable to hydrologic drought. Affected households obtain water from neighborhood reservoirs, wells, or home cisterns. During drought periods, these reservoirs either dry up or become heavily polluted, and cisterns that help maintain regular water supplies during the wet season cannot withstand long dry periods. In central drought regions, farmers become heavily dependent on government water trucks.

Scientists, politicians, and the public are now faced with the challenge of developing adaptive responses to climate change. Several studies predict a decline in reservoir water availability in NEB due to climate change (Araújo et al. 2004; Krol et al. 2011). Societies must now adapt to impending scenarios.

There are several ways of examining how NEB ecosystem characteristics limit growth. In natural conditions without perennial rivers or reservoirs for water transport during high discharge and dry years, such limits are negligible. Narratives of droughts expelling Aboriginal populations from sertões in 1583 and of the great drought (1877–1879) that killed roughly half a million people serve as proof of such limitations.

Reservoir construction (*hydraulic mission*) has increased the region’s hydrological potential, elevating the environmental limit. This is the threshold referred by Duque (1953).

Hydrological resource management activated during hydraulic missions will introduce environmental limits modified by human kind. Effective management will be associated with social justice and sustainability. On the other hand, poor management will aggravate drought impacts and pollute water resources, thus proving unsustainable.

Water management for desertification-prone environment resilience will prove challenging in the future. To cope up with this challenge, it is necessary introduce a new conceptual framework that is based on green–blue water and on the identification of “hydrological opportunities in a sea of constraints” (Falkenmark and Rockström 2008).

## Final Remarks

This historical review shows that changes in public drought policies have not been characterized by abrupt shifts, but rather by consistency and robustness in ways that politicians, technical personnel, scientists, and societies as a whole have implemented policy solutions to drought problems in NEB.

Debates concerning the misuse of public resources have accompanied drought relief programs since 1877. This is characteristic of most programs that involve significant public resources. The issue is occasionally discussed in regional disputes over federal resources. Appeals for corruption are directly proportional to the amount of public money implicated, and inversely proportional to the transparency of money application. Hence, while drought programs attract corruption, the potential for misuse depends on the way in which funds are applied, and not on drought policies themselves.

Brazilian society has been struggling to manage drought impacts for a long period of time. Fortunately, this struggle has been alleviated, as drought impacts on the rural NEB population have been greatly reduced. For the MWS, available reservoir infrastructure renders the NEB less vulnerable to hydrologic drought. For the FWS, which is still highly vulnerable, social programs focus on conditional money transfers. Currently, scientists must determine how climate change will affect social and physical systems that have been in place for over a century. Researchers are currently attempting to answer such questions; nevertheless, there is still much work to be done.

Public debates surrounding drought are commonplace in the media (i.e., during the 2012–2013 drought). However, now paradigms are constructed from ideas voiced in international forums such as the World Water Congress and United Nations Millennium Declaration. The World Water Congress will be held in Brazil in 2018, and drought, public policy, and poverty issues will undoubtedly feature in discussions.

NEB ecosystem limits placed on growth can be examined in various ways. In natural conditions without perennial rivers and reservoirs to transport water during high discharge and dry years, such limits are negligible. Narratives of droughts expelling Aboriginal populations from sertões in 1583 of the great drought (1877–1879) that killed roughly half a million people serve as a proof of these limitations.

The hydraulic mission of NEB reservoir construction has elevated the region’s environmental limit. Water management practices will thus affect the impact of environmental modification by humans. While effective water management policies will consider principles of social justice and sustainability, poor water management strategies will aggravate drought impacts and pollute waters, creating an unsustainable environment. Finally, in generating knowledge on the environment, proposing water resource management strategies and by debating alternative management schemes, scientists can help the general public better identify sustainable practices.

## Footnotes

1 Available at www.brasiliana.usp.br.

2 Data from the Brazilian Institute of Geography and Statistics (IBGE). Available at: http://www.ibge.gov.br/home/estatistica/populacao/censohistorico/default_hist.shtm.

3 While participating as a consultant in the formulation of the PERH in 1992, I noted that water markets were discussed in several debates. Nevertheless, the local community did not accept such propositions.
